# Validation of survey information on smoking and alcohol consumption against import statistics, Greenland 1993–2010

**DOI:** 10.3402/ijch.v72i0.20314

**Published:** 2013-03-05

**Authors:** Peter Bjerregaard, Ulrik Becker

**Affiliations:** 1 Centre for Health Research in Greenland, National Institute of Public Health, University of Southern Denmark, Odense, Denmark; 2 Department of Medical Gastroenterology, Copenhagen University Hospital, Hvidovre, Denmark

**Keywords:** alcohol consumption, tobacco consumption, binge drinking, validity, population surveys, Greenland, Inuit

## Abstract

**Background:**

Questionnaires are widely used to obtain information on health-related behaviour, and they are more often than not the only method that can be used to assess the distribution of behaviour in subgroups of the population. No validation studies of reported consumption of tobacco or alcohol have been published from circumpolar indigenous communities.

**Objective:**

The purpose of the study is to compare information on the consumption of tobacco and alcohol obtained from 3 population surveys in Greenland with import statistics.

**Design:**

Estimates of consumption of cigarettes and alcohol using several different survey instruments in cross-sectional population studies from 1993–1994, 1999–2001 and 2005–2010 were compared with import statistics from the same years.

**Results:**

For cigarettes, survey results accounted for virtually the total import. Alcohol consumption was significantly under-reported with reporting completeness ranging from 40% to 51% for different estimates of habitual weekly consumption in the 3 study periods. Including an estimate of binge drinking increased the estimated total consumption to 78% of the import.

**Conclusion:**

Compared with import statistics, questionnaire-based population surveys capture the consumption of cigarettes well in Greenland. Consumption of alcohol is under-reported, but asking about binge episodes in addition to the usual intake considerably increased the reported intake in this population and made it more in agreement with import statistics. It is unknown to what extent these findings at the population level can be inferred to population subgroups.

Questionnaires are widely used to obtain information on health-related behaviour, and they are more often than not the only method that can be used to assess the distribution of behaviour in subgroups of the population. Self-reported smoking has been validated in several populations, and generally a very high agreement between self-report and measurements of urine cotinine has been found (1–3) although a study among Australian Aboriginals showed a fairly low agreement with number of cigarettes smoked (4).

Self-reporting of alcohol consumption is less precise. Young men in Australia fairly accurately estimated their consumption 1–2 days after single episodes of drinking as long as the amount consumed was small or moderate but underestimation increased with heavy consumption (5). In a sample of adult men and women from Denmark, simple questions about the typical alcohol intake for each day of the week corresponded well to the results of a 7-day recall and gave somewhat higher figures than asking about average weekly intake, but results were not compared with an external assessment (6). In another study from Denmark, there was a negative correlation between the recall period and self-reported alcohol intake while asking about beverage-specific intake instead of overall alcohol intake increased the reported amount of alcohol (7). Stockwell and co-workers (8) found under-reporting of alcohol consumption in self-completed questionnaires in an Australian population and recommended the use of both recent recall and measures of longer term drinking patterns as well as detailed questions about the size of drinks consumed instead of relying on standard drinks. Including a measure of binge drinking in the calculation of average daily alcohol consumption substantially increased the estimated prevalence of heavy drinkers in the US (9).

No validation studies of reported consumption of tobacco or alcohol have been published from circumpolar indigenous communities. In Greenland, import statistics for tobacco and alcohol are available since 1975. The purpose of the paper is at the population level to compare information on the consumption of tobacco and alcohol obtained from population surveys in Greenland in 1993–1994, 1999–2001 and 2005–2010 with import statistics from the same years.

## Methods

The total population of Greenland is 57,000 of whom 90% are ethnic Greenlanders (Inuit). Genetically, Greenlanders are Inuit (Eskimos) with a mixture of European, mainly Scandinavian genes. They are genetically and culturally closely related to the Inuit/Iñupiat in Canada and Alaska and, somewhat more distantly, to the Yupiit of Alaska and Siberia.

### Data collection

Data were collected by interview and self-administered questionnaires as part of 3 general population health surveys. In 1993–1994, data (N=1728) were collected in 38 towns and villages in Greenland as part of a general population health survey with a participation rate of 57% (10). In 1999–2001, data (N=1936) were collected in 7 towns and villages on the west coast of Greenland as part of a general population health survey with a participation rate of 67% (11). In 2005–2010, data (N=2958) were collected in 21 towns and villages as part of a general population health survey with a participation rate of 67% (12). Questionnaires were developed in the Danish language, translated into Greenlandic, back-translated and revised. The questions were originally adapted from a Danish health interview survey for the 1993–1994 study (10, 13) and subsequently developed with the inclusion of questions from surveys among the Inuit in Canada and Alaska. Interviews gave information about socio-demographic factors, self-rated health and disease, and lifestyle including diet, physical activity and smoking. Information about alcohol use, among other topics, was obtained from self-administered questionnaires. Interviews were conducted in the language of choice of the participant, most often in Greenlandic, by native Greenlandic speaking interviewers who had been trained in the study procedures. The questionnaires were available in Greenlandic and Danish. In all 3 surveys, some or all participants participated in a clinical survey of cardiovascular risk factors, environmental health and other issues.

### Variables

Information about the import of cigarettes and alcohol was obtained from Statistic Greenland's online database (14) for the years corresponding to the surveys. Figures for tobacco include cigarettes and cigarette paper. Figures for alcohol include local production of beer but exclude tax-free purchases in international airports and home production. Survey information about smoking was obtained from questions on smoking status (current smoker, past smoker, never smoker) and number of cigarettes smoked per day. The use of other tobacco products including smokeless tobacco is infrequent in Greenland and was not studied.

Four different survey instruments were used to estimate alcohol consumption (Table I). The last occasion instrument consisted of 1 question about when the respondent last had a drink and 1 question about the number of drinks consumed on the last occasion. The habitual weekly days instrument consisted of 2 questions about how many days per week the respondent usually drank alcohol (specific number of days or if not available the following frequency categories: daily/almost daily; 3–6 times per week; 1–2 times a week; less often) and 1 question about the number of drinks consumed on the last occasion. The beverage-specific instrument consisted of 3 questions about habitual weekly consumption of beer, wine and strong liquor. The answers were added to give the number of drinks consumed per week. The combined weekly days and binge instrument was the sum of the habitual weekly days consumption and an estimate of the amount consumed on binge episodes. The latter was calculated from a question on frequency of binge drinking multiplied by the reported population average of drinks consumed at a binge episode (9.4 drinks). A standard drink corresponds to 12 g of pure alcohol corresponding to 1 beer, 1 glass of table wine or 3 cl of 40% proof spirit or 15 ml of pure alcohol. In import statistics, yearly consumption is expressed in litres of 100% alcohol per person aged 15+ years. In order to compare reported drinks with import statistics, the number of drinks per week was multiplied by 52 and divided by the population aged 15+ to give the yearly consumption per adult and further multiplied by 0.015.

**Table I:** Distribution of response to questions about alcohol consumption, Greenland 1993–2010

Question	Response categories	Studies	N	Valid %
Weekly drinking frequency				
When did you last have a beer or a glass of wine or liquor?		1993–2010	5757	%
	Today or yesterday		409	7.6
	During the last week		2018	37.6
	During the last month		1001	18.7
	More than 1 month ago		831	15.5
	No alcohol last 12 months		1106	20.6
	Missing information (7%)		392	
How many days a week do you drink alcohol?		1999–2010	4364	%
	0		1347	41.2
	1		1091	33.4
	2		438	13.4
	3		121	3.7
	4		49	1.5
	5		67	2.1
	6		38	1.2
	7		116	3.6
	Missing information (25%)		1105	
How often do you drink beer, wine or liquor?		1999–2010	4364	%
	Daily or almost daily		60	1.4
	3–6 times per week		156	3.7
	1–2 times per week		757	17.8
	1–3 times per month		1039	24.5
	Less often		1359	32.0
	No alcohol last 12 months		876	20.6
	Missing information (3%)		117	
How many drinks do you usually drink per week?		1999–2001	1838	mean
	Beer		1167	5.0
	Wine		1168	0.9
	Liquor		1165	0.8
	Missing information (36%)		670	
Binge frequency				
In the past 12 months, how often have you had 5 or more drinks on the same occasion (same evening, same party, etc.)		2005–2010	2526	%
	More than once a week		125	5.5
	Once a week		267	11.7
	2–3 times a month		413	18.0
	Once a month		284	12.4
	Less than once a month		492	21.5
	Never		154	6.7
	No alcohol last 12 months		556	24.3
	Missing information (9%)		235	
Amount consumed				
How much did you drink on that occasion? (last time)		1993–2010	5757	%
	1 beer or glass of wine or liquor		547	10.6
	2–5 beer or glasses of wine or liquor		1333	25.8
	6–10 beer or glasses of wine or liquor		1370	26.6
	More than 10 beer or glasses of wine or liquor		802	15.5
	No alcohol last 12 months		1106	21.4
	Missing information (10%)		599	

N=5757 Inuit who answered a self-administered questionnaire.

### Statistical methods

Statistical analyses were performed in IBM SPSS version 20 (15). In Table II, estimates of survey based alcohol consumption were age and sex adjusted to the population mean by the Univariate procedure of SPSS (General Linear Model). Estimation of population total consumption was performed in Excel from age-, sex- and ethnicity-specific consumption. For the study in 1999–2001 where all participants were Inuit, non-Inuit were assumed to have the same age- and sex-specific consumption as Inuit.

**Table II:** Drinks per week estimated by different survey instruments in 3 population surveys in Greenland (N=5475; Inuit only)

	Survey instrument				
	
	Last occasion	Habitual weekly days	Beverage specific	Weekly +Binge	Import statistics
	Drinks per week	Drinks per week	Drinks per week	Drinks per week	Drinks per week
1993–1994	7.3				17.2
1999–2001	7.0	7.3	6.7		17.2
2005–2010	6.1	8.2		11.8	15.1
p	<0.001	0.04			<0.001

Adjusted for age and sex in General Linear Models and compared with import statistics.

### Ethical considerations

The studies were ethically approved by the Commission for Scientific Research in Greenland. Participants gave their oral (1993–1994) or written consent after being informed about the study orally and in writing.

## Results

The questions on tobacco were posed by interviewers to all participants and 98% answered. The questions on alcohol were posed in a self-administered questionnaire in which 91% answered, while a further 95% of these answered the questions about alcohol consumption (87% of all participants; 58% of the sample). There were small but statistically significant differences between the studies with a higher response rate in the 1999–2001 study. The study base thus consisted of 6517 participants with information on tobacco and 5757 with information on alcohol. Of these, 6223 and 5475, respectively, were Inuit (95%).

**Fig. 1:**
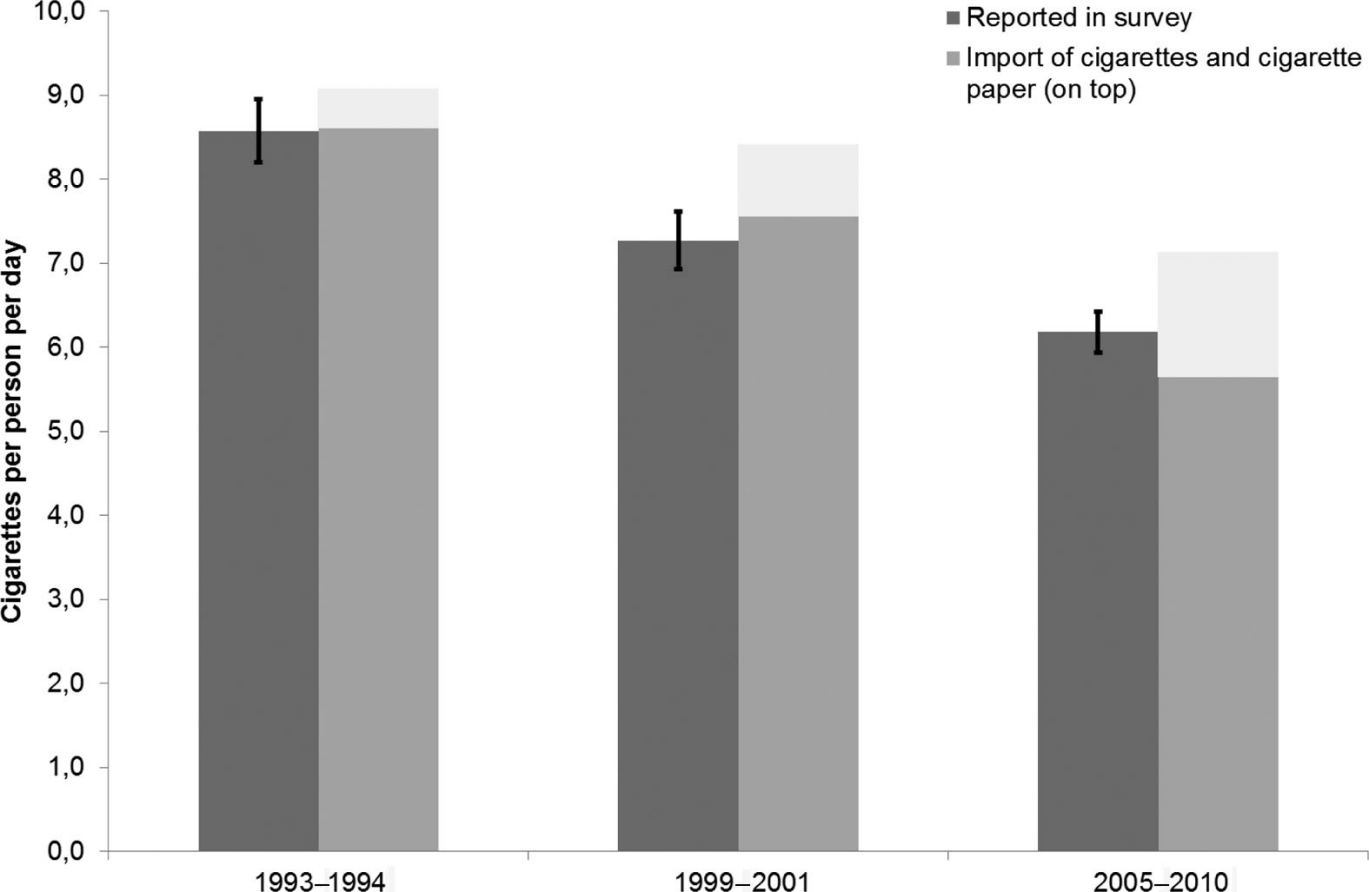
Self-reported consumption of cigarettes and import of cigarettes and cigarette paper, Greenland 1993–2010.

The import of cigarettes decreased considerably from 1993 to 2010 as did the reported consumption of cigarettes while the import of cigarette paper increased. Fig. 1 shows that the survey results accounted for virtually the total import.

Four different survey instruments were used to estimate the consumption of alcohol, 1 in 1993–1994, 3 in 1999–2001 and 3 in 2005–2010 (Tables I and II). Within the same study, the estimates varied with the lowest estimate for the beverage-specific method and the highest reported consumption from the combined weekly days and binge method. For the last occasion estimation method, which was applicable to all 3 studies, the average reported consumption decreased from 7.3 to 6.1 drinks per week during the period studied (p<0.001) while it increased between the 2 last surveys by the weekly days method.

For the 2005–2010 survey, we compared participants in the first 2 weeks of data collection with those of the last 2 weeks because based on the impression of the interviewers, we hypothesized that unstable socioeconomic position and alcohol-related behaviour increased over the survey weeks. Although there was a general tendency in support of the hypothesis, the difference was only statistically significant in 1 of 5 towns where due to the survey procedures it could be tested.

**Fig. 2:**
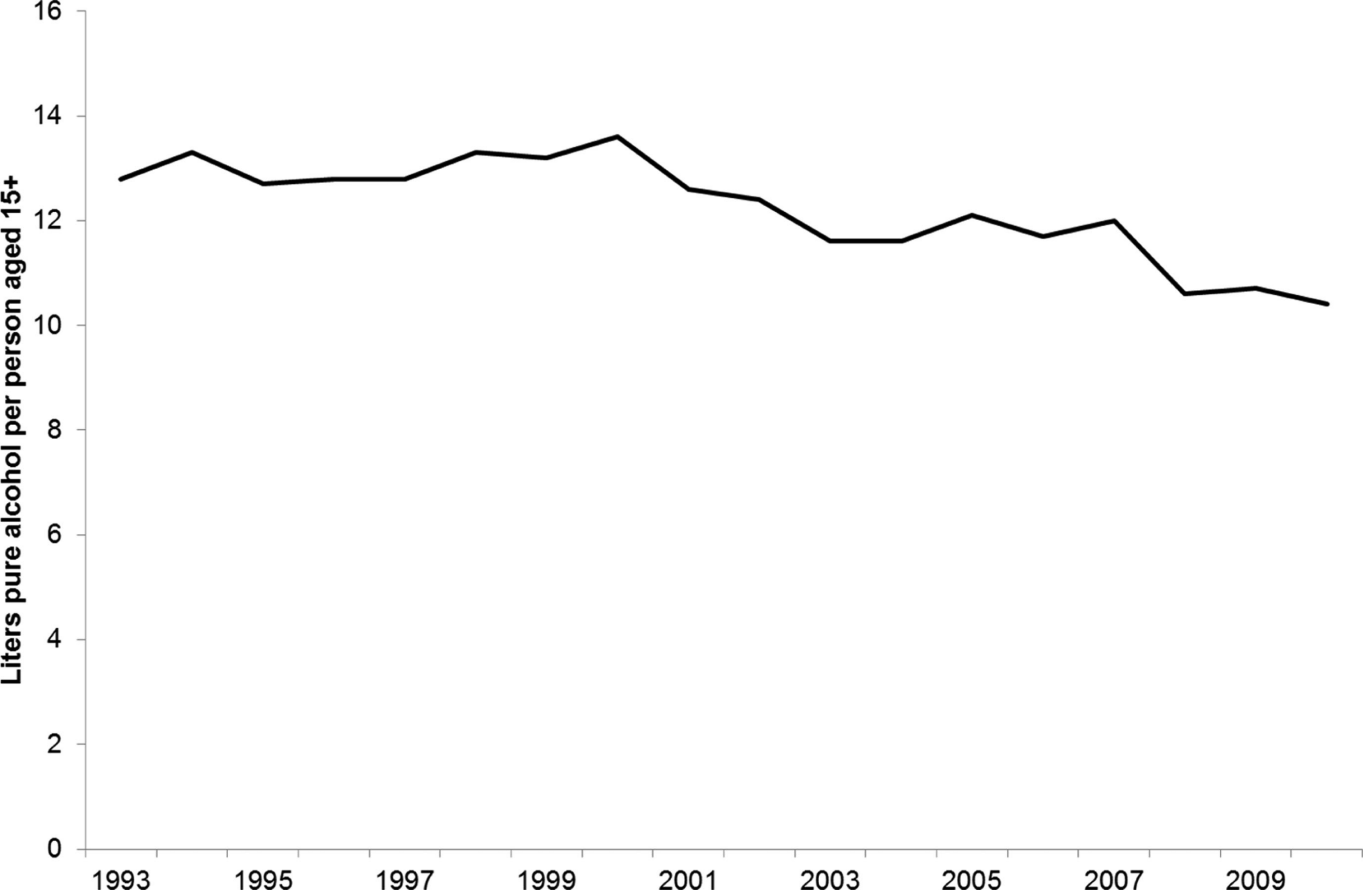
Import of alcohol to Greenland 1993–2010. Source: Statistics Greenland.

**Fig. 3:**
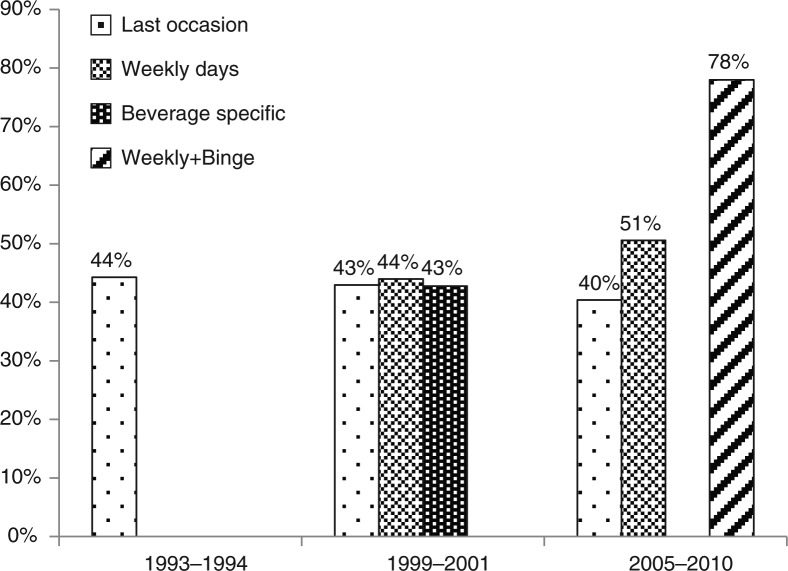
Self-reported consumption of alcohol by 4 different survey instruments as a percent of import, Greenland 1993–2010.

In 1993–1994, the import was equivalent to 13.1 litres of 100% alcohol per person aged 15+; in 1999–2001, 13.1 litres; and in 2005–2010, 11.5 litres (Fig. 2). Figure 3 compares the reported alcohol consumption for the whole population with the import statistics. The combined weekly days and binge method (weekly+binge) performed well and was able to account for 78% of the import, while the other survey methods were able to account for 40–50% of the import only. It is noteworthy that for the last occasion method, which has figures for all 3 surveys, reported percentage did not change much over the years, i.e. 44% in 1993–1994, 43% in 1999–2001 and 40% in 2005–2010. Accordingly, the decreasing import was reflected in the surveys although the absolute level was grossly under-reported. The reported consumption of alcohol thus followed the import fairly closely with a decrease of 80% from 1993–1994 to 2005–2010 for the last occasion instrument and 88% for import. It is also remarkable that in the 1999–2001 study, the estimates by all 3 methods used were very similar, which was not the case in the 2005–2010 study although the methods used were the same.

## Discussion

As in studies from other countries, we found a very good accordance between self-report and an objective measures for tobacco (in our case import statistics) but a moderate to poor accordance for alcohol. It is a strength of the study that the surveys were countrywide, giving information from a large Inuit population. Although import statistics and calculations of reported consumption were for the entire population, 90% of the population in Greenland is Inuit and the results can therefore, with a reasonable certainty, be extrapolated to Inuit.

For tobacco, the main uncertainty is the proportion of cigarette paper used for rolling traditional cigarettes. Since the import of cigarette paper relative to the import of cigarettes increased from 6% in 1994 to 23% in 2010, this could be a source of bias when comparing the validity of reporting in the 3 studies.

There could be a number of reasons for the under-reporting of alcohol consumption. It is a weakness of the study that only 58% of the sample reported their alcohol consumption. It is well known that non-responders have a higher average alcohol intake and a higher prevalence of alcohol-related diagnoses (16). Furthermore, non-responders had a lower income than participants at least in the 2005–2010 study (12), and there was a strong association between low income or wealth and high alcohol consumption in all 3 studies (own unpublished results), which suggests a significant under-reporting due to social participation bias. In a Canadian study, adjusting for non-response increased the estimated percentage of persons with heavy alcohol use by just 4% (17). This could be more pronounced in Greenland. Furthermore, those who reported their alcohol intake might underestimate their average intake by not taking binge episodes into considerations (9), an issue that was addressed by the weekly+binge measure apparently with some success. Stockwell et al. (8) argued that poor recall of alcohol consumption and inability to make accurate estimates of mean intake on drinking days played a role. Another possible confounding factor is under-reporting due to the stigma attached to heavy drinking (5), which may play a role even in self-administered questionnaires.

In all 3 studies, questions about alcohol use were included in a confidential self-administered questionnaire along with other sensitive topics. A study among US undergraduate students revealed that there were no differences in disclosure rates due to methods for questions on a series of sensitive topics (18). In Greenland, only 91% of participants filled in the self-administered questionnaire; one of the reasons given by participants was that they had forgotten their reading glasses, but the percentage did not increase much after the participants were offered reading glasses. It was the impression of the staff that a certain degree of functional illiteracy could be the reason and the inclusion of the questions about alcohol in the interview could therefore potentially increase participation rates for this topic. On the other hand, assuming similar relaxed attitudes to sensitive questions among a population sample of adult Inuit and a sample of US undergraduate students is at most tentative. A change from the self-administered questionnaire to an interview could lower the response rate for the alcohol questions and introduce a risk of additional reporting bias.

For calculations, we used a standard drink size of 12 g (15 ml) pure alcohol. This is equivalent to the alcohol contents of a normal beer in Greenland, and since 70% of the alcohol imported to Greenland is beer, the bias from variation in the size of drinks is presumably small unlike the findings of Stockwell et al. (8).

Import statistics are probably a very good measure of consumption in Greenland. Some relatively small amounts of alcohol should ideally be added to the import statistics due to tax-free import, but other cross border purchase is not possible due to Greenland's isolated location. Based on passenger statistics and the legally permitted amounts of tax-free import, a liberal estimate of tax-free import would be equivalent to 0.5 drinks per week. However, this is partly balanced by the consumption by visitors. Home production of beer, wine and alcohol is illegal, and its magnitude is unknown but according to the police it plays a small role (19). A study from Svalbard, where like in Greenland the registered import of alcohol is a valid estimate of the total availability, showed similar results to our study, i.e. a self-report of 40% of the sales volume (20).

Among the 4 survey instruments used the weekly+binge method resulted in the highest estimate of alcohol consumption and accordingly the best correlation with import statistics. However, only one data point was available (for the 2005–2010 study). The other 3 survey instruments showed similar correlations with import statistics, but there were many missing values for the beverage-specific method (36%). Also, the question on weekly number of days with alcohol consumption had many missing values and had to be supplemented by a more crude categorical question.

With the present data, it was possible to estimate total consumption over a 17-year period by applying a conversion factor of 2.3–2.5 to the reported consumption (last occasion method). In evaluating these results, it is important to bear in mind that data on the association between alcohol intake and health risks in many instances also have been calculated on the basis of underestimated alcohol intake. Therefore, these risk functions as well as sensible drinking limits are probably still valid. However, import statistics are only published at country level, and it is therefore not possible to compare the validity of reported consumption among population groups within Greenland or among high and low consumers. Future research should focus on improving the survey instruments and testing them in subgroups of the population in relation to age, sex, region and social position.

## Conclusion

Compared with import statistics, questionnaire-based population surveys capture the consumption of cigarettes well in Greenland. Consumption of alcohol is under-reported, but asking about binge episodes in addition to usual intake considerably increased the reported intake in this population and made it in better accordance with import statistics. It is unknown to what extent these findings at the population level can be inferred to population subgroups.
